# JSH practical guidelines for hematological malignancies, 2023: II. lymphoma—overview

**DOI:** 10.1007/s12185-025-03920-6

**Published:** 2025-03-04

**Authors:** Ken Ohmachi

**Affiliations:** Department of Internalmedicine, Division of Hematology and Oncology, Tokai University School of Medicine, 143 Shimokasuya, Isehara-City, Kanagawa 259-1143 Japan

**Keywords:** Lymphoma, JSH practical guideline

The number of patients with newly diagnosed lymphoma in Japan was reported to be 35,782 in 2018. The prevalence has been increasing over the years, from 5.5 per 100,000 population in 1985 to 8.9 in 1995, 13.3 in 2005, and 28.3 in 2018. It is more common in men than women (ratio of about 3:2) and incidence peaks in the 70–80 age group [1].

## Essential elements for diagnosis and treatment plan determination

1) Medical history

The patient’s past medical history, diseases for which they are currently being treated, comorbidities, initial symptoms, timing of symptom onset, general symptoms (e.g., fever, weight loss, and night sweats) , family history of hematological malignancies, and place of birth if necessary, should be determined by medical interview and recorded.

2) Physical findings

The following should be evaluated by physical examination and recorded:

- Height, weight, body temperature, blood pressure, pulse
- Performance status
- Anemia and jaundice, skin rash, auscultation and percussion of chest and abdomen, lymph node enlargement (and sites [region names and laterality], number, size, and properties [e.g., stiffness and mobility] if enlarged lymph nodes are present), palpable hepatomegaly/splenomegaly, edema
- Motor nerve paralysis, paresthesia, and meningeal signs

3) General tests

The following tests should be performed:

- Complete blood count, morphology (white blood cell count, neutrophil count, lymphocyte count, tumor cell count, red blood cell count, hemoglobin level, platelet count, morphology)
- Erythrocyte sedimentation rate (used for Hodgkin lymphoma [HL])
- Blood biochemistry (TP, Alb, ALT, AST, LDH, ALP, γ-GTP, Na, K, Cl, Ca, P, BUN, Cr, FBS, UA)
- Serological tests (CRP, IgG, IgA, IgM, protein fractions, soluble IL-2R, beta-2 microglobulin [essential for follicular lymphoma, may be useful in some cases of mantle cell lymphoma and diffuse large B-cell lymphoma])
- Virus tests (HBs antigen, HBs antibody [Ab], HBc Ab, HCV Ab, HIV Ab, HTLV-1 Ab)
- Urinalysis (glucose, protein, occult blood, sediment)
- Imaging and other tests (chest X-ray, 12-lead electrocardiography, contrast [when possible] computed tomography [CT] of the neck/chest/abdomen/pelvis, ^18^F-fluorodeoxyglucose positron emission tomography (FDG-PET), upper and lower gastrointestinal endoscopy [as needed], bone marrow aspiration or biopsy, echocardiography, and, as needed, head CT or magnetic resonance imaging [MRI], cerebrospinal fluid analysis, and arterial blood gas analysis)

4) Histopathological diagnosis

Histopathological examination by biopsy is generally essential to the diagnosis of lymphoma and biopsy of the appropriate lesion must be performed before treatment. As the inguinal lymph nodes can enlarge reactively, biopsy of the cervical lymph nodes is preferable in patients with generalized lymphadenopathy. Histopathological examination by needle biopsy alone is often insufficient for diagnosis, and thus open biopsy should be performed when feasible.

Biopsy specimens should be processed into formalin-fixed paraffin blocks, cut into thin sections, and stained with hematoxylin and eosin. Other immunohistochemical tests such as those listed below, as well as Epstein–Barr encoding region (EBER) in situ hybridization, should also be performed.

CD45, cytoplasmic CD3ε, CD5, CCR4, CD20, CD79a, CD10, immunoglobulin (cytoplasmic immunoglobulin), CD56, CD15, CD30, cyclin D1, BCL2, BCL6, MIB1 (Ki-67), IRF4/MUM1, MYC, ALK, etc.

5) Other tests

Tumor cells should be isolated from the specimen as best as possible and the following tests performed:

- Flow cytometry
- Chromosome analysis
- Fluorescence in situ hybridization (e.g., BCL2, BCL6, MYC, CCND1, and MALT1)
- Gene analysis (e.g., immunoglobulin rearrangements, T-cell receptor rearrangements, and *EZH2*)

## Classification of the lymphoid neoplasms

The 2017 WHO classification is widely used as a classification of lymphoma. Lymphoid neoplasms, which include lymphoma, are classified as follows: [2, 3]

Mature B-cell neoplasms

Chronic lymphocytic leukemia/small lymphocytic lymphoma

Monoclonal B-cell lymphocytosis

Monoclonal B-cell lymphocytosis CLL-type

Monoclonal B-cell lymphocytosis non CLL-type

B-cell prolymphocytic leukemia

Splenic marginal zone lymphoma

Hairy cell leukemia

Splenic B-cell lymphoma/leukemia, unclassifiable

Splenic diffuse red pulp small B-cell lymphoma

Hairy cell leukemia variant

Lymphoplasmacytic lymphoma

Waldenström macroglobulinemia

IgM monoclonal gammopathy of undetermined significance

Heavy-chain disease

Mu heavy-chain disease

Gamma heavy-chain disease

Alpha heavy-chain disease

Plasma cell neoplasms

Non-IgM monoclonal gammopathy of undetermined significance

Plasma cell myeloma

Plasma cell myeloma variants

Smoldering (asymptomatic) plasma cell myeloma

Non-secretory myeloma

Plasma cell leukemia

Plasmacytoma

Solitary plasmacytoma of bone

Extraosseous plasmacytoma

Monoclonal immunoglobulin deposition disease

Primary amyloidosis

Light-chain and heavy-chain deposition diseases

Plasma cell neoplasms with associated paraneoplastic syndrome

POEMS syndrome

TEMPI syndrome

Extranodal marginal zone lymphoma of mucosa-associated lymphoid tissue (MALT lymphoma)

Nodal marginal zone lymphoma

Pediatric nodal marginal zone lymphoma

Follicular lymphoma

Testicular follicular lymphoma

In situ follicular neoplasia

Duodenal-type follicular lymphoma

Pediatric-type follicular lymphoma

Large B-cell lymphoma with IRF4 rearrangement

Primary cutaneous follicle center lymphoma

Mantle cell lymphoma

Leukemic non-nodal mantle cell lymphoma

In situ mantle cell neoplasia

Diffuse large B-cell lymphoma (DLBCL), NOS

Germinal center B-cell subtype

Activated B-cell subtype

T-cell/histiocyte-rich large B-cell lymphoma

Primary diffuse large B-cell lymphoma of the CNS

Primary cutaneous diffuse large B-cell lymphoma, leg type

EBV-positive diffuse large B-cell lymphoma, NOS

EBV-positive mucocutaneous ulcer

Diffuse large B-cell lymphoma associated with chronic inflammation

Fibrin-associated diffuse large B-cell lymphoma

Lymphomatoid granulomatosis

Primary mediastinal (thymic) large B-cell lymphoma

Intravascular large B-cell lymphoma

ALK-positive large B-cell lymphoma

Plasmablastic lymphoma

Primary effusion lymphoma

HHV8-associated lymphoproliferative disorders

Multicentric Castleman disease

HHV8-positive diffuse large B-cell lymphoma, NOS

HHV8-positive germinotropic lymphoproliferative disorder

Burkitt lymphoma

Burkitt-like lymphoma with 11q aberration

High-grade B-cell lymphoma

High-grade B-cell lymphoma, with MYC and BCL2 and/or BCL6 rearrangement

High-grade B-cell lymphoma, NOS

B-cell lymphoma, unclassifiable, with features intermediate between DLBCL and classic Hodgkin lymphoma

Mature T-cell and NK-cell neoplasms

T-cell prolymphocytic leukemia

T-cell large granular lymphocytic leukemia

Chronic lymphoproliferative disorder of NK cells

Aggressive NK-cell leukemia

EBV-positive T-cell and NK-cell lymphoproliferative diseases of childhood

Systemic EBV-positive T-cell lymphoma of childhood

Chronic active EBV infection of T- and NK-cell type, systemic form

Hydroa vacciniforme-like lymphoproliferative disorder

Severe mosquito bite allergy

Adult T-cell leukemia/lymphoma

Extranodal NK/T-cell lymphoma, nasal type

Intestinal T-cell lymphoma

Enteropathy-associated T-cell lymphoma

Monomorphic epitheliotropic intestinal T-cell lymphoma

Indolent T-cell lymphoproliferative disorder of the gastrointestinal tract

Hepatosplenic T-cell lymphoma

Subcutaneous panniculitis-like T-cell lymphoma

Mycosis fungoides

Sézary syndrome

Primary cutaneous CD30 positive T-cell lymphoproliferative disorders

Lymphomatoid papulosis

Primary cutaneous anaplastic large cell lymphoma

Primary cutaneous peripheral T-cell lymphomas, rare subtypes

Primary cutaneous gamma delta T-cell lymphoma

Primary cutaneous CD8-positive aggressive epidermotropic cytotoxic T-cell lymphoma

Primary cutaneous acral CD8-positive T-cell lymphoma

Primary cutaneous CD4+ small/medium T-cell lymphoproliferative disorder

Peripheral T-cell lymphoma, NOS

Angioimmunoblastic T-cell lymphoma and other nodal lymphomas of T follicular helper (TFH) cell origin

Angioimmunoblastic T-cell lymphoma

Follicular T-cell lymphoma

Nodal peripheral T-cell lymphoma with TFH phenotype

Anaplastic large cell lymphoma, ALK-positive

Anaplastic large cell lymphoma, ALK-negative

Breast implant-associated anaplastic large cell lymphoma

Hodgkin lymphoma

Nodular lymphocyte predominant Hodgkin lymphoma

Classic Hodgkin lymphoma

Nodular sclerosis classic Hodgkin lymphoma

Lymphocyte-rich classic Hodgkin lymphoma

Mixed-cellularity classic Hodgkin lymphoma

Lymphocyte depleted classic Hodgkin lymphoma

## Clinical categorization

The Working Formulation proposed in 1982 not only classifies non-Hodgkin lymphoma (NHL) into disease entities but also categorizes NHL by grade based on its natural history. If left untreated, low-grade NHL progresses over a matter of years, intermediate-grade over a matter of months, and high-grade over a matter of weeks. In 1989, the United States National Cancer Institute proposed a clinical categorization in which low-grade lymphomas are categorized as indolent, intermediate-grade as aggressive, and high-grade as highly aggressive to expand categorization beyond grade to aggressiveness, which reflects characteristics such as malignant nature, activity, and invasiveness. This categorization has been widely used in clinical trials. WHO classification entities roughly fall under the following clinical categorizations: [4]

Indolent lymphoma.

B cell

Chronic lymphocytic leukemia/small lymphocytic lymphoma

Lymphoplasmacytic lymphoma

Splenic marginal zone lymphoma

Extranodal marginal zone lymphoma of mucosa-associated lymphoid tissue (MALT lymphoma)

Nodal marginal zone lymphoma

Follicular lymphoma

Mantle cell lymphoma

T cell

T-cell large granular lymphocytic leukemia

Adult T-cell leukemia/lymphoma (smoldering, some chronic)

Mycosis fungoides/Sezary syndrome

Primary cutaneous anaplastic large cell lymphoma

Aggressive lymphoma

B cell

Diffuse large B-cell lymphoma

T cell

Peripheral T-cell lymphoma, not otherwise specified

Enteropathy-associated T-cell lymphoma

Anaplastic large cell lymphoma

Hepatosplenic T-cell lymphoma

Adult T-cell leukemia/lymphoma (acute, lymphomatous, some chronic)

Extranodal NK/T-cell lymphoma, nasal type

Angioimmunoblastic T-cell lymphoma

Highly aggressive lymphoma

B cell

Burkitt lymphoma/leukemia

T cell

Aggressive NK-cell leukemia

## Staging systems

The extent of lymphoma greatly affects treatment selection and prognosis, which makes accurate staging critical. General staging of lymphoma is determined by medical history, physical findings, complete blood count and morphology, blood biochemistry, chest X-ray, CT of the neck/chest/abdomen/pelvis, and, as necessary, upper and lower gastrointestinal endoscopy and bone marrow aspiration or biopsy.

FDG-PET/CT has recently been adopted for staging of lymphoma. As FDG avidity differs between histologic types of lymphoma, when FDG-PET/CT will be used for response assessment, it should ideally also be used for pre-treatment staging to ensure more accurate assessment of the significance of lesions.

The Ann Arbor classification, which was developed for staging of HL, has also been used for NHL (Table 1) [5], and the 2014 Lugano classification was created at the International Conference on Malignant Lymphoma as a modified version of the Ann Arbor classification (Table 2) [6]. In the 2014 Lugano classification, stage is determined by FDG-PET/CT before treatment for lymphomas that show high FDG uptake if FDG-PET/CT will be used for response assessment. General A and B symptoms do not need to be recorded for NHL, and bone marrow biopsy is not necessary for HL and diffuse large B-cell lymphoma if FDG-PET/CT has been performed.

**Table 1:** Ann Arbor classification

Stage I	Involvement of a single lymph node region (I); or localized involvement of a single extralymphatic organ or site in the absence of any lymph node involvement (IE)
Stage II	Involvement of two or more lymph node regions on the same side of the diaphragm (II); or localized involvement of a single extralymphatic organ or site in association with regional lymph node involvement with or without involvement of other lymph node regions on the same side of the diaphragm (IIE) The number of involved regions may be written in subscript, for example, as II_3_
Stage III	Involvement of lymph node regions on both sides of the diaphragm (III), which also may be accompanied by extralymphatic extension in association with adjacent lymph node involvement (IIIE) or by involvement of the spleen (IIIS) or both (IIIES)
Stage IV	Diffuse or disseminated involvement of one or more extralymphatic organs, with or without associated lymph node involvement; or isolated extralymphatic organ involvement in the absence of adjacent regional lymph node involvement, but in conjunction with disease in distant site(s)

A and B classification (symptoms)

Each stage is classified as A or B based on general symptoms as defined below

1) Fever: Unexplained changed. fever > 38 °C

2) Night sweats: Drenching sweat so intense that the bed linens (including all bed coverings besides the mattress [e.g., comforter and sheets] but not sleepwear) must be

3) Weight loss: Unexplained loss of > 10% of usual body weight within the 6 months preceding diagnosis

From Reference [5]

**Table 2:** 2014 Lugano classification (modified staging system for primary nodal lymphomas)

Stage		Involvement	Extranodal status (E)
Limited	Stage I	One node or group of adjacent nodes	Single extranodal lesion without nodal involvement
	Stage II	Two or more nodal groups on the same side of the diaphragm	Stage I or II by nodal extent with limited, contiguous extranodal involvement
	Stage II bulky*	Stage II as above with bulky disease	N/A
Advanced	Stage III	Nodes on both sides of the diaphragm, or nodes above the diaphragm with spleen involvement	N/A
	Stage IV	Non-contiguous extranodal involvement in addition to nodal lesions	N/A

Disease progression is assessed by PET for lymphomas that show uptake and by CT for those that do not show uptake. Tonsils, Waldeyer’s ring, and spleen are considered nodal tissue

^*^Whether stage II bulky disease is treated as limited or advanced disease may be determined by histology and number of prognostic factors. As the definition of bulky disease differs by histology, the longest diameter should be recorded rather than using the “X” notation

The A and B classification (symptoms) of the Ann Arbor classification was removed from the 2014 Lugano classification. They are only added for Hodgkin lymphomaReprinted with permission from Cheson BD, et al., Recommendations for Initial Evaluation, Staging, and Response Assessment of Hodgkin and Non-Hodgkin Lymphoma: The Lugano Classification, J Clin Oncol. 2014; 32(27): 3059–3067

(https://ascopubs.org/doi/10.1200/JCO.2013.54.8800); Copyright ⓒ 2014 American Society of Clinical Oncology

As the main lesion is extranodal in primary gastrointestinal lymphoma, the Ann Arbor stage is often inconsistent with actual disease progression in such lymphomas. Therefore, the 1994 Lugano staging system for primary gastrointestinal lymphoma created at the International Conference on Malignant Lymphoma is used in addition to the Ann Arbor classification (Table 3) [7].

**Table 3:** Lugano staging system for primary gastrointestinal lymphoma (1994)

Stage I	Confined to gastrointestinal tract Single primary, or multiple non-contiguous lesions
Stage II	Extending into abdomen from primary gastrointestinal site Nodal involvement II_1_: Local (perigastric if gastric lymphoma or peri-intestinal if intestinal lymphoma) II_2_: Distant (mesenteric if primary intestinal, or paracaval, paraaortic, pelvic, or inguinal if not)
Stage IIE	Penetration of serosa to involve adjacent organ or tissues. Specify site of involvement, e.g., IIE_[pancreas]_, IIE_[large intestine]_, IIE_[retroperitoneum]_ If both nodal involvement and involvement of adjacent organs, denote stage using both a subscript (1 or 2) and E, e.g., II_1_ E_[pancreas]_
Stage IV	Disseminated extranodal involvement or gastrointestinal tract lesion with supra-diaphragmatic nodal involvement

From Reference [7]

## Prognostic factors and prognostic model

Lymphoma is broadly categorized histologically into two prognostic groups: low-grade and intermediate-to-high-grade. Various other factors besides histology, including molecular category, stage, and individual patient characteristics such as performance status, are known to influence prognosis as well. The International Prognostic Index (IPI) is used as a prognostic model for aggressive lymphoma (Table 4) [8]. Recently, the National Comprehensive Cancer Network IPI (NCCN-IPI) has been proposed as a prognostic index for diffuse large B-cell lymphoma based on outcomes after the introduction of rituximab (Table 5) [9]. Although the Follicular Lymphoma International Prognostic Index (FLIPI) (Table 6) [10] was used for follicular lymphoma, it was a prognostic model for overall survival created using data from retrospective studies conducted before the introduction of rituximab. FLIPI2 was later created as a predictive model of progression-free survival using data from prospective studies conducted after the introduction of rituximab (Table 7) [11]. The International Prognostic Score (IPS) is used as a prognostic model for advanced Hodgkin lymphoma (Table 8) [12].

**Table 4:** Aggressive lymphoma: IPI

Prognostic factors in IPI	Unfavorable prognostic factors
Age	> 60 years
Serum LDH	> upper limit of normal
Performance status	2 to 4
Stage	III or IV
Number of involved extranodal sites	≥ 2

From Reference [8]

Patients are classified into the following four risk groups by number of prognostic factors:

0 or 1 factor: low risk

2 factors: low–intermediate risk

3 factors: high–intermediate risk

4 or 5 factors: high risk

**Table 5:** Diffuse large B-cell lymphoma: NCCN- IPI

Unfavorable prognostic factors in NCCN-IPI	Score
Age	
41 to 60 years	1
61 to 75 years	2
> 75 years	3
Serum LDH	
Above but within three times the upper limit of normal	1
Over three times the upper limit of normal	2
Stage III or IV	1
Extranodal involvement (bone marrow, central nervous system, liver/gastrointestinal tract, lungs)	1
Performance status ≥ 2	1

From Reference [9]

Patients are classified into the following four risk groups by their score:

Score of 0 or 1: low risk

Score of 2 or 3: low–intermediate risk

Score of 4 or 5: high–intermediate risk

Score of 6 or higher: high risk

**Table 6:** Follicular lymphoma: FLIPI

Prognostic factors in FLIPI	Unfavorable prognostic factors
Age	> 60 years
Serum LDH	> upper limit of normal
Hemoglobin level	< 12 g/dL
Number of nodal sites involved	≥ 5 sites
Stage	III or IV

From Reference [10]

Patients are classified into the following three risk groups by number of prognostic factors:

0 or 1 factor: low risk

2 factors: intermediate risk

3 or more factors: poor risk

**Table 7:** Follicular lymphoma: FLIPI2

Prognostic factors in FLIPI2	Unfavorable prognostic factors
Age	> 60 years
Beta-2 microglobulin level	> upper limit of normal
Hemoglobin level	< 12 g/dL
Longest diameter of largest involved node	> 6 cm
Bone marrow involvement	Yes

Data from Federico M, et al.: J Clin Oncol 27(27), 2009: 4555–4562

Readers are encouraged to read the entire article for the correct context at jco.ascopubs.org

Patients are classified into the following three risk groups by number of prognostic factors:

0 factors: low risk

1 or 2 factors: intermediate risk

3 or more factors: high risk

**Table 8:** Advanced Hodgkin lymphoma: IPS

Prognostic factors in IPS	Unfavorable prognostic factors
Serum albumin level	< 4 g/dL
Hemoglobin level	< 10.5 g/dL
Sex	Male
Age	≥ 45 years
Stage	IV
White blood cell count	≥ 15,000/mm^3^
Lymphocytes	< 600/mm^3^, or < 8% fraction of white blood cells

From Reference [12]

The IPS is defined as the number of prognostic factors

## Response criteria

CT is typically used in response assessment for lymphoma, and the 2014 Lugano classification was published (Table 9) [6] after research suggested that FDG-PET/CT was widely used and clinically useful. These response criteria were created as an effort toward global standardization of clinical trial evaluation, but treatment response assessed by FDG-PET/CT is better correlated with prognosis than response assessed by CT alone, which makes FDG-PET/CT useful for response assessment in routine clinical practice as well. It is recommended to use the five-point scale (5PS) when assessing treatment response by FDG-PET/CT (Table 10) [13].

**Table 9:** Revised response criteria in the 2014 Lugano classification

Response and site	PET-CT-based response	CT-based response
Complete	Complete metabolic response	Complete radiologic response (all of the following)
Lymph nodes and extralymphatic sites	Score 1, 2, or 3* with or without a residual mass on 5PS It is recognized that in Waldeyer’s ring or extranodal sites with high physiologic uptake or with activation within spleen or marrow (e.g., with chemotherapy or myeloid colony-stimulating factors), uptake may be greater than normal mediastinum and/or liver. In this circumstance, complete metabolic response may be inferred if uptake at sites of initial involvement is no greater than surrounding normal tissue even if the tissue has high physiologic uptake	Target nodes/nodal masses must regress to ≤ 1.5 cm in longest diameter No extralymphatic sites of disease
Non-measured lesions	N/A	Absent
Organ enlargement	N/A	Regress to normal
New lesions	None	None
Bone marrow	No evidence of FDG-avid disease in marrow	Normal by morphology; if indeterminate, immunohistochemistry negative
Partial	Partial metabolic response	Partial remission (all of the following)
Lymph nodes and extralymphatic sites	Score 4 or 5 on 5PS with reduced uptake compared with baseline and residual mass(es) of any size. At interim, these findings suggest responding disease At end of treatment, these findings indicate residual disease	≥ 50% decrease in the sum of the product of the perpendicular diameters of up to 6 target nodes and extranodal sites When a lesion is too small to measure on CT, assign 5 mm × 5 mm as the default value When no longer visible, 0 mm × 0 mm For a node > 5 mm × 5 mm, but smaller than normal, use actual measurement for calculation
Non-measured lesions	N/A	Absent/normal, regressed, but no increase
Organ enlargement	N/A	Spleen must have regressed by > 50% in length beyond normal
New lesions	None	None
Bone marrow	Residual uptake higher than uptake in normal marrow but reduced compared with baseline (diffuse uptake compatible with reactive changes from chemotherapy allowed) If there are persistent focal changes in the marrow in the context of a nodal response, consideration should be given to further evaluation with MRI or biopsy or an interval scan	N/A
No response or stable disease	No metabolic response	Stable disease
Target nodes/nodal masses Extranodal lesions	Score 4 or 5 with no significant change in FDG uptake from baseline at interim or end of treatment	< 50% decrease in the sum of the product of the perpendicular diameters of up to 6 dominant, measurable nodes and extranodal sites No criteria for progressive disease are met
Non-measured lesions	N/A	No increase consistent with progression
Organ enlargement	N/A	No increase consistent with progression
New lesions	None	None
Bone marrow	No change from baseline	N/A
Progressive disease	Progressive metabolic disease	Progressive disease meeting at least 1 of the following:
Individual target nodes/nodal masses Extranodal lesions	Score 4 or 5 with an increase in intensity of uptake from baseline and/or new FDG-avid foci consistent with lymphoma at interim or end-of-treatment assessment	An individual node/lesion must be abnormal with: Longest diameter > 1.5 cm and increase by ≥ 50% from the nadir of the cross product of the perpendicular diameters Increase in longest diameter or shortest diameter from nadir (0.5 cm for lesions ≤ 2 cm, 1.0 cm for lesions > 2 cm) In the setting of splenomegaly, the splenic length must increase by > 50% of the extent of its prior increase beyond baseline (e.g., a 15-cm spleen must increase to > 16 cm) If no prior splenomegaly, must increase by at least 2 cm from baseline New or recurrent splenomegaly
Non-measured lesions	None	New or clear progression of preexisting non-measured lesions
New lesions	New FDG-avid foci consistent with lymphoma rather than another etiology (e.g., infection, inflammation) If uncertain regarding etiology of new lesions, biopsy or interval scan may be considered	Regrowth of previously resolved lesions A new node > 1.5 cm in any axis A new extranodal site > 1.0 cm in any axis If < 1.0 cm in any axis, its presence must be unequivocal and must be attributable to lymphoma Assessable disease of any size unequivocally attributable to lymphoma
Bone marrow	New or recurrent FDG-avid foci	New or recurrent involvement

^*^ In many patients, a score of 3 indicates a good prognosis with standard treatment, especially if at the time of an interim scan. However, in trials involving PET where de-escalation is investigated, it may be preferable to consider a score of 3 as inadequate response (to avoid undertreatment)

**Table 10:** Five-point scale

1	No uptake
2	Uptake below mediastinum
3	Uptake above mediastinum, but below or equal to uptake in the liver
4	Uptake slightly to moderately higher than the liver
5	Markedly higher uptake than liver, and/or appearance of any new lesion
X	New area of uptake not overtly attributable to lymphoma

Adapted with permission from Barrington SF, et al., Role of Imaging in the Staging and Response Assessment of Lymphoma: Consensus of the International Conference on Malignant Lymphomas Imaging Working Group, J Clin Oncol. 2014; 32(27): 3048–3058. (https://ascopubs.org/doi/10.1200/JCO.2013.53.5229); Copyright Ⓒ 2014 American Society of Clinical Oncology

Measured dominant lesions: Select up to six of the largest dominant nodes, nodal masses, and extranodal lesions that are clearly measurable in two diameters. Nodes should preferably be from disparate regions of the body and should include, where applicable, mediastinal and retroperitoneal areas. Non-nodal lesions include those in solid organs (e.g., liver, spleen, kidneys, lungs), GI involvement, cutaneous lesions, or those noted on palpation.

Non-measured lesions: Any disease not selected as measured, dominant disease and truly assessable disease should be considered not measured. These sites include any nodes, nodal masses, and extranodal sites not selected as dominant or measurable or that do not meet the requirements for measurability but are still considered abnormal, as well as truly assessable disease, which is any site of suspected disease that would be difficult to follow quantitatively with measurement, including pleural effusions, ascites, bone, meningeal, and abdominal lesions, abnormal lesions, and other lesions that cannot be confirmed and followed by imaging.

In Waldeyer’s ring or in extranodal sites (e.g., GI tract, liver, bone marrow), FDG uptake may be greater than in the mediastinum with complete metabolic response, but should be no higher than surrounding normal physiologic uptake (e.g., with marrow activation as a result of chemotherapy or myeloid growth factors).

Reprinted with permission from Cheson BD, et al., Recommendations for Initial Evaluation, Staging, and Response Assessment of Hodgkin and Non-Hodgkin Lymphoma: The Lugano Classification, J Clin Oncol. 2014; 32(27): 3059–3067. (https://ascopubs.org/doi/10.1200/JCO.2013.54.8800); Copyright Ⓒ 2014 American Society of Clinical Oncology.

## Post-treatment follow-up

Methods of post-treatment follow-up and evaluation differ by disease type and by setting (i.e., clinical trial versus routine clinical practice). Performing a complete blood count, blood biochemistry, and imaging appropriately, alongside careful history-taking and examinations, is important in making appropriate clinical decisions.

Although there is no evidence to provide clear guidelines regarding the frequency and duration of follow-up, it is recommended to perform follow-up assessments for Hodgkin lymphoma and curable aggressive lymphoma every 2 to 3 months for the first 2 years after a complete response and at least every 3 to 6 months for 3 years after that point. It is recommended to perform follow-up assessments for indolent lymphoma that is unlikely to be cured, every 2 to 3 months for the first year after treatment and then every 3 to 6 months thereafter.

Previous studies reported that relapse of lymphoma is detected by clinical symptoms in over 80% of cases [14, 15]. Although relapse can sometimes be detected before appearance of clinical symptoms by regular CT scans, no clear evidence indicates whether early detection improves prognosis [16]. Because CT is more widely used in Japan than in other countries, CT scans are relatively accessible. This could explain why the estimated cumulative risk of cancer by diagnostic X-ray up to age 75 years in Japan is reported to be more than 3%, nearly twice as high as figures in Western countries [17]. CT is an extremely useful test for staging, response assessment, and diagnosis of relapse in lymphoma. However, unnecessary testing exposes patients to unnecessary radiation and increases cancer risk. Regular follow-up by FDG-PET/CT is not recommended due to a lack of evidence of any benefit [18–21]. Studies have also shown that routine blood tests also have limited utility for detecting early progression or relapse of asymptomatic indolent lymphoma, aggressive lymphoma in first remission, or Hodgkin lymphoma in first remission [22–24]. Regular follow-up imaging and blood tests should only be performed when the risks (including toxicity and cost) and benefits to the patient have been weighed and the benefits are judged to outweigh the risks.
